# MRI contrast accumulation in features of cerebral small vessel disease: blood-brain barrier dysfunction or elevated vascular density?

**DOI:** 10.1186/s12987-025-00675-4

**Published:** 2025-07-16

**Authors:** Tomas Vikner, Anders Garpebring, Cecilia Björnfot, Jan Malm, Anders Eklund, Anders Wåhlin

**Affiliations:** 1 Department of Diagnostics and Intervention, Umeå University, Umeå, SE S-90187 Sweden; 2 Department of Applied Physics and Electronics, Umeå University, Umeå, SE S-90187 Sweden; 3 Department of Medical Physics, School of Medicine and Public Health, University of Wisconsin-Madison, Madison, WI 53792 USA; 4 Umeå Center for Functional Brain Imaging (UFBI), Umeå University, Umeå, S-90187 Sweden; 5 Department of Clinical Science, Neurosciences, Umeå University, Umeå, S-90187 Sweden

**Keywords:** MRI, Small vessel disease, White matter lesions, Perivascular spaces, Blood-brain barrier

## Abstract

**Background:**

White matter lesions (WML) and dilated perivascular spaces (PVS) are features of small vessel disease (SVD), commonly observed in aging and dementia, with unknown pathophysiology. Human studies have documented contrast accumulation within and in proximity of SVD-lesions. However, whether such observations mainly reflect excessive blood-brain barrier (BBB) leakage, or altered microvascular density in the investigated regions, remains unclear.

**Methods:**

To evaluate the roles of BBB leakage and vascular density in aging and SVD, dynamic contrast enhanced (DCE) MRI was used to estimate the permeability-surface area product (PS) and fractional plasma volume ($\:{v}_{p}$) in normal-appearing brain tissue and in proximity of and within WML and PVS in a population-based cohort (*N* = 56; 34/22 m/f; age 64 to 84 years). Analysis of variance (ANOVA) was used to assess regional differences in PS and $\:{v}_{p}$ and analysis of covariance (ANCOVA) was used to assess regional differences in PS with $\:{v}_{p}$ and vascular risk as covariates.

**Results:**

Pronounced increases in PS and $\:{v}_{p}$ were observed from normal-appearing white matter (NAWM) to WML peripheries to WMLs. Similar PS and $\:{v}_{p}\:$increases were observed from basal ganglia (BG) to BG-PVS. Further, PS in NAWM and white matter (WM) PVS were found to increase with cortex-to-ventricular depth. However, ANCOVA models with $\:{v}_{p}$ as a covariate showed that variance in PS was mainly explained by v_p_ (η^2^=0.17 to η^2^=0.35; all *p* < 10^− 3^), whereas the effect of region was only borderline-significant when comparing NAWM, WML peripheries and WML (*p* = 0.03) and non-significant for the other comparisons (*p* > 0.29).

**Conclusions:**

Our findings support the notion that contrast leakage across the BBB accumulates within and in proximity of SVD-related lesions. However, high contrast accumulation may mainly reflect high vascularization, and to a lesser degree than previously recognized BBB dysfunction.

**Supplementary Information:**

The online version contains supplementary material available at 10.1186/s12987-025-00675-4.

## Background

Cerebral small vessel disease (SVD) is a common cause of vascular dementia (VaD) and a comorbidity of Alzheimer’s disease (AD) [1]. White matter lesions (WML) and dilated perivascular spaces (PVS) are commonly assessed as radiological features associated with age, vascular health, and cerebral SVD [2, 3]. However, multiple cerebrovascular age- and dementia-related changes often progress relatively concomitantly. Currently, there are no available curative treatment options for SVD, other than anti-hypertensive agents [4]. This is partially due to an incomplete understanding of the pathophysiology, highlighting the need to identify imaging biomarkers that develop early in the pathogenesis [2] that could lead to novel prevention and treatment strategies.

Several hypotheses exist regarding the etiology of WML progression and PVS dilation [2, 5]. However, WML and PVS volumes often correlate [6] and increasing evidence suggests that SVD is a diffuse whole-brain disease that affects the white matter (WM) of the entire brain [7]. This points towards a shared underlying pathophysiology in WML and PVS progression, and blood-brain barrier (BBB) disruption could be an initial pathological event [8]. The BBB consists of endothelial cells, maintained by capillary pericytes, that protects the brain by assuring that large molecules and toxic substances are retained in the bloodstream [9]. In experimental studies, pericyte-deficient mice develop BBB breakdown, PVS dilation, and WML-related axonal injury [10]. Hence, animal studies suggest that diffuse white matter (WM) damage, including subtle BBB leakages, likely precedes the development of more pronounced vascular lesions associated with SVD [10].

Human studies using dynamic contrast enhanced (DCE) MRI have shown that BBB leakages in normal-appearing WM (NAWM) increase with proximity to WMLs in patients with VaD [11]. Further, recent studies have assessed the spatial distribution of WMLs using a combination of lobar parcellation and WM depth (cortex-to-ventricles distance [12]), and linked WML patterns to cognitive impairment [13] and AD [14]. Such findings suggest that periventricular WM could be particularly susceptible to vascular damage. Moreover, BBB leakage in NAWM, WMLs, cortex, and deep grey matter were related to SVD burden among elderly (49 to 90 years) free from cerebrovascular and neurodegenerative disease [15]. In the same cohort, higher BG-PVS leakage was related to PVS severity [16]. Yet, leakage comparisons between PVS and healthy tissue are lacking. Furthermore, studies attempting to elucidate the role of BBB dysfunction in aging and SVD typically use Patlak analysis to quantify the permeability-surface area product (PS) or $\:{K}_{trans}$ to describe the rate of contrast accumulation across the BBB [17–19]. However, PS reflects the product of BBB permeability (P) and microvascular surface area (S) [18]. Hence, studies quantifying PS (or $\:{K}_{trans}$) in isolation, without considering the potential confounding effects of vascularization patterns and vascular density with or in proximity of vascular lesions, could lead to false interpretations of the underlying pathophysiology.

To evaluate the role of microvascular dysfunction in WML and PVS progression, we use DCE MRI to quantify PS and fractional plasma volume (V_p_) in an elderly population-based cohort. Specifically, we compare PS and $\:{v}_{p}$ among several regions of interest (ROIs), including NAWM, WMLs, WML peripheries, WM PVS, basal ganglia (BG), and BG-PVS. Further, we evaluate PS and $\:{v}_{p}$ in NAWM and PVS as a function of WM depth (normalized cortex-to-ventricle distance). Finally, we follow up our analyses of regional PS-differences with $\:{v}_{p}$ as a covariate (and a proxy of vascular surface area), to isolate the effects of ROI and vascular density on BBB leakage.

## Materials and methods

### Participants

The study population consisted of elderly individuals (*N* = 61; age 68 to 84 years), randomly invited from the population registry in Umeå, Sweden. Exclusion criteria included compromised kidney function and allergic reactions to medical treatment, or any other contraindication for intravenous contrast. Before the MRI session, participants were asked about various medical conditions and medications, had their blood pressure measured in the left arm during seated position, and underwent a Montreal Cognitive Assessment (MoCA) test where all participants scored at least 20/30 points. Results from the health assessment are described in Table 1. After the MRI session, two participants (pilot scans) were excluded due to problems with the T1 maps (technical errors), and another three participants were excluded due to problems with the contrast injections where less than one third of the group-averaged contrast was detected in the venous system. Hence, a total of *N* = 56 participants were included. The study was conducted in accordance with the Declaration of Helsinki and approved by the national ethical review authority (dnr: 2020–03710). Written informed consent was provided from all participants prior to the experiments.

**Table 1:** Characteristics of the cohort (*N* = 56)

Health parameter		Mean (SD)
Age (years)		76.3 (3.9)
Sex (men/women)		34/22
Body mass index		25.1 (3.7)
Diabetes mellitus		7
Hypertension		33
Hyperlipidemia		29
Atrial fibrillation		11
Transient ischemic attack		2
Stroke		4
SBP (mmHg)		132 (24)
DBP (mmHg)		73.8 (13)
MoCA score		25.8 (4.2)
WML volume (ml)		4.35 (3.85)
WM PVS volume (ml)		9.33 (3.08)
BG PVS volume (ml)		0.60 (0.30)
Intracranial volume (ml)		1552 (165)

Note: SBP/DBP, systolic/diastolic blood pressure; MoCA, Montreal Cognitive Assessment; WM, white matter; WML, white matter lesions; BG, basal ganglia; PVS, perivascular space, SD, standard deviation

### Magnetic resonance imaging

MRI scans were performed using a 3 Tesla (Discovery MR 750; GE Healthcare, Milwaukee, Wisconsin) and a 32-channel head coil, and included an anatomical T1 weighted scan for ROI extraction and lesion segmentation, and a T1 weighted series for T1 mapping and DCE series to analyze Gadolinium leakage across the BBB.

Anatomical T1 weighted scans were acquired with a 3D fast spoiled gradient echo (FSPGR) sequence and the following parameters: TR/TE 8.2/3.2 ms, flip angle 12$\:^\circ\:$, 176 slices with 1 mm thickness, in-plane resolution 0.94 mm, field of view 25 × 25 cm [2] and a phase acceleration of 2.

The dynamic contrast enhanced (DCE) MRI sequence included a series (60 frames) of T1 weighted FSPGR scans, acquired over a total time of 17.5 min with a temporal resolution of 17.5 s. After about one minute into the scan, intravenous Gadolinium contrast (Dotarem; 0.1 mmol/kg) was administered using a slow injection (lasting for about one minute). The following imaging parameters were used for the DCE sequence: TR/TE 4.5/1.6 ms, flip angle 12$\:^\circ\:$, slice thickness 1.5 mm, in-plane resolution 0.82 mm, field of view 21 × 21 over 108 slices, partial Fourier 6/8, and parallel imaging factor 2.

The variable flip angle (VFA) method and a set of three T1-weighted FSPGR scans, all obtained with the same imaging parameters as the DCE sequence but flip angles 2, 8, and 12$\:^\circ\:$, were used for T1 mapping. Two frames were acquired for each flip angle and averaged before the T1 estimation.

B1 mapping was used to correct for deviations and spatial inhomogeneities in the achieved compared to the indented flip angle, using the Bloch-Siegert shift approach [20] and the following parameters: flip angle 15$\:^\circ\:$, slice thickness 5 mm, and in-plane resolution 3.91 mm.

### White matter, basal ganglia, and white matter lesion segmentation

Tissue probability maps for GM, WM, and cerebrospinal fluid (CSF) were automatically segmented using SPM12. NAWM, WML, and BG (caudate, putamen, and pallidum), were segmented using FreeSurfer 6.0. In addition, WML peripheries were defined by dilation of the WML region (3 mm spherical kernel), multiplication with the NAWM region, and subtraction of the WML region. Finally, an adjusted WML ROI was defined by excluding voxels with a CSF probability > 0.05 or a T1 time > 4000 ms to minimize potential CSF contamination of the WML concentration curves.

### Perivascular spaces segmentation

**Fig. 1:**
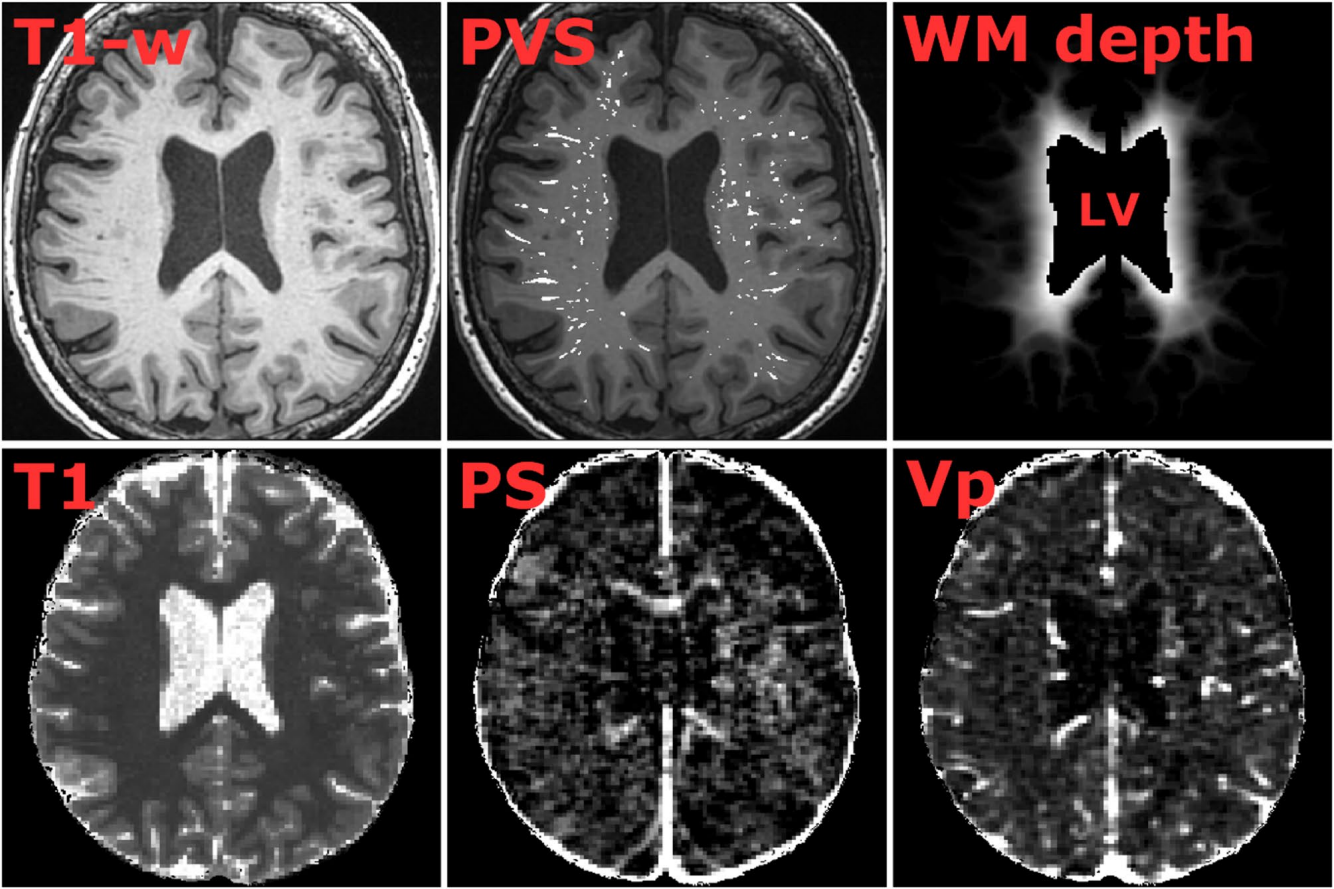
Anatomical T1-weighted MRI, segmented perivascular spaces (PVS), normalized cortex-ventricular white matter (WM) depth, baseline T1 map, permeability-surface area product (PS), and fractional plasma volume ($\:{v}_{p}$) for an example participant. Quantitative visualizations range from zero to a maximum of 5s (T1), 2.5$\:\cdot\:$10^−3^$\:\cdot\:$ min^− [1]^ (PS), and 5% ($\:{v}_{p}$). Note that ROI-averaged concentration curves were used to estimate PS and $\:{v}_{p}$ for all statistical analyses, whereas these parameter maps were intended for visualization only

WM- and BG-PVS were segmented from high-resolution T1-weighted scans (0.49 × 0.49 × 1.00 mm [3]). First, a hessian-based filter (Jerman et al. [21]) designed to boost the intensity of tubular/vascular structures was used to improve PVS visualization. Next, a binary mask including both WM and BG was defined from the FreeSurfer output and eroded using a spherical kernel (1 mm radius) to exclude artifacts, boundaries, and other structures of no interest from the filtered T1-weighted volume. Global intensity-based thresholds were applied to the filtered volume (Fig. 1; Sup. Figure 1) to obtain WM PVS (5% threshold) and BG PVS (15% threshold) segmentations for the main analyses. In addition, PVS were segmented for multiple thresholds (5–25% in 1% steps) to evaluate the sensitivity of the main variables (volume, PS, and $\:{v}_{p}$) to the segmentation to procedure. While no manual PVS count were performed in the current study, we previously evaluated automatic PVS segmentation against visual scores (according to Potter et al. [22]), with *r* = 0.79 for PVS volume against PVS count in the semioval center [6].

### White matter depth

WM depth (or distance) maps were computed by estimating the normalized distance from the cortex to the ventricles, inspired by a bullseye parcellation of cerebral WM [12]. The distance to the cortex ((dctx) and to the ventricles (dvent) was estimated voxel-wise for all NAWM voxels (MATLAB bwdist). Normalized distances maps were then estimated from cortex to ventricles as $\:{d}_{CV}={d}_{ctx}/{(d}_{ctx}+{d}_{vent})$ such that all values ranged from zero to one (Fig. 1; Sup. Figure 2). The depth maps were segmented into three depth regions, including near-cortical, intermediate, and periventricular WM segments to analyze NAWM and PVS PS and $\:{v}_{p}$ as a function of WM depth. The three regions were defined (individually) such that each WM depth contained equally many voxels.

### DCE MRI processing and gadolinium concentration derivation

Gadolinium contrast concentration curves (mM) were calculated using the T1 maps and DCE series as input. The T1-weighted scans for the DCE series and T1 mapping were initially pre-processed with a filter to suppress Gibbs ringing artifacts [23]. The filtered data were then co-registered to the anatomical T1-weighted volume using a two-step approach: a rigid-body registration followed by a boundary-based registration as implemented in FreeSurfer (V6.0) suite [24]. The rest of the DCE and BBB leakage analyses were performed in MATLAB R2022b (Natick, Massachusetts: The MathWorks Inc) using in-house tools.

T1 maps were calculated using the variable flip angle (VFA) approach [25] from the T1 weighted scans acquired with three flip angles (2, 8, and 12$\:^\circ\:$) while also considering B1 map correction. Specifically, corrected flip angle maps, defined as the intended flip angles (2, 8, and 12$\:^\circ\:$) multiplied by the B1 map, were used as input to the VFA method. B1 correction was similarly applied to correct flip angle deviations in the DCE series data. From the DCE series and the T1 maps, concentration curves were estimated assuming fast water exchange rates [26] according to:

$$\:C\left(t\right)=\frac{1}{{r}_{1}}\left({R}_{1}\left(t\right)-{R}_{10}\right),$$

where $\:{r}_{1}$ is the relaxivity of the contrast agent (3.5 L$\:\cdot\:$mmol^−1^s^− [1]^ in blood plasma [27]), $\:{R}_{10}=\frac{1}{{T}_{10}}$ is the relaxation rate at baseline, and $\:{R}_{1}\left(t\right)$ is the relaxation rate as a function of time (or concentration), estimated from the DCE time curves and T1 maps, as described in detail by Schabel et al. [26]

### Blood-brain barrier leakage analysis

The Patlak model was used to estimate permeability-surface area product (PS), describing the leakage rate, and the fractional plasma volume, $\:{v}_{p}$, assuming unidirectional (irreversible) flow from the BBB to the extracellular fluid [18]. The tracer concentration $\:{C}_{tissue}$ was modelled as:

$$\:{C}_{tissue}\left(t\right)=PS{\int\:}_{0}^{t}{C}_{VIF}\left(\tau\right)d\tau+{v}_{p}{C}_{VIF}\left(t\right).$$

where $\:{C}_{VIF}$ is the vascular input function (VIF) concentration extracted in the superior sagittal sinus (SSS) to minimize bias from sensitive to motion artifacts, partial volume errors, and inflow effects [18]. Specifically, the VIF concentration was calculated as:

$$\:{C}_{VIF}\left(t\right)=\frac{{C}_{b}\left(t\right)}{1-HCT},$$

where $\:{C}_{b}$ is the contrast concentration in blood as estimated from the T1 maps and DCE series, and a hematocrit (HCT) of 0.45 was assumed [17]. The VIF concentration curves were extracted by averaging along a 25 voxel venous centerline in the posterior part of the SSS, as previously evaluated by two raters [28]. The Patlak equation was solved for PS and $\:{v}_{p}$ by multiple linear regression, using ROI-averaged tissue concentration curves ($\:{C}_{tissue}$) as input. Example T1, PS, and $\:{v}_{p}$ are shown in Fig. 1. The extended Tofts (or modified Tofts) model [17] was also used to assess PS, $\:{v}_{p}$ and extracellular volume fraction ($\:{v}_{e}$) to compare PS and $\:{v}_{p}$ between models.

### Statistics

**Fig. 2:**
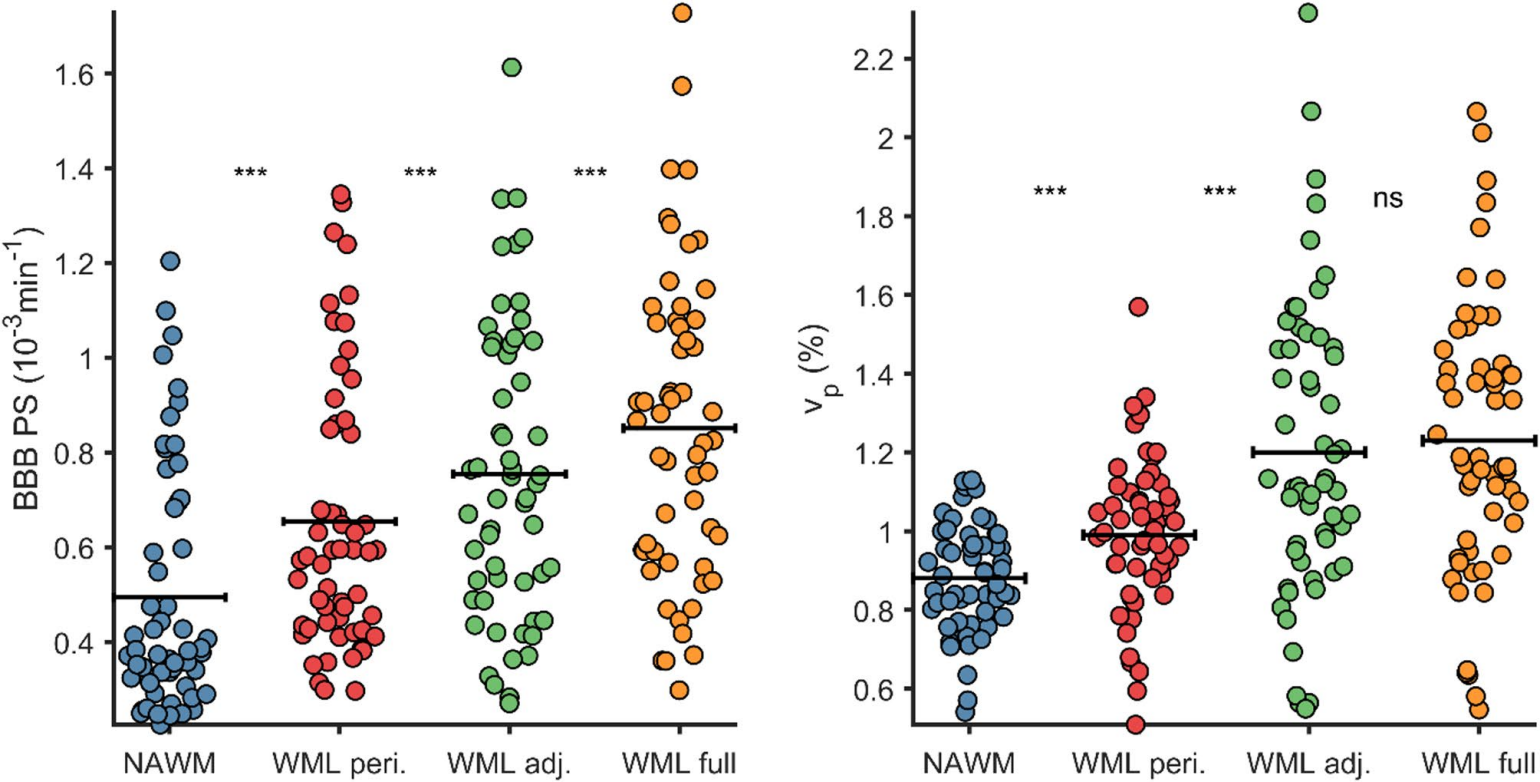
Increased permeability-surface area product (PS) going from normal-appearing white matter (NAWM) to white matter lesion (WML) peripheries to WMLs, including WML adjusted for CSF probability (< 5%) and T1 time at baseline (> 4000ms). Similar increases were also observed for vascular fraction ($\:{v}_{p}$). ***p < 0.001 and ns (non-significant) from paired t-tests (multiple comparison adj.). Statistical differences were also obtained using repeated measures ANOVA for PS (F = 101; *p* < 0.001) and $\:{v}_{p}$ (F = 53; *p* < 0.001)

**Fig. 3:**
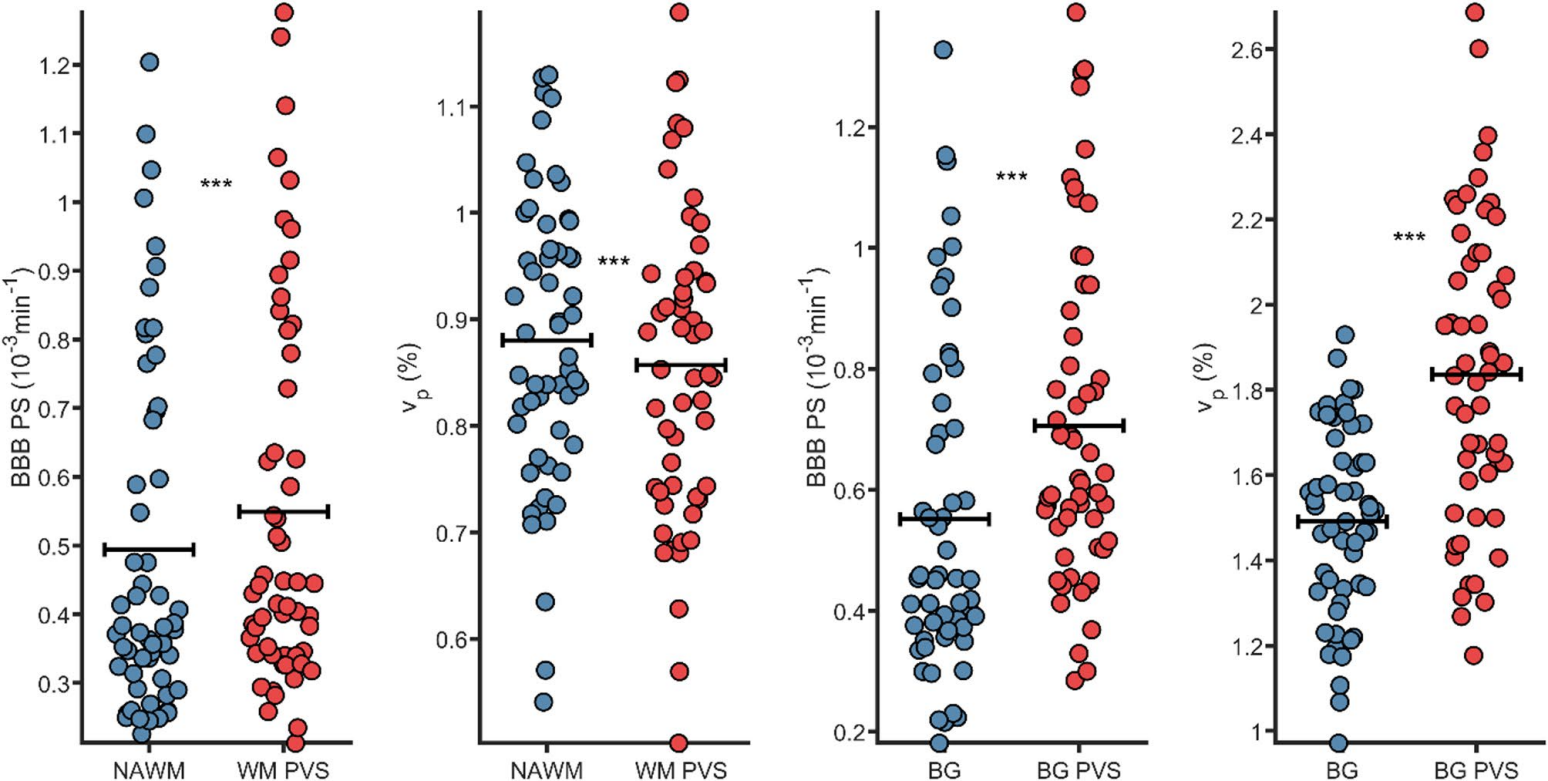
Permeability-surface area product (PS) and fractional plasma volume ($\:{v}_{p}$) from Patlak analysis in normal-appearing white matter (NAWM), basal ganglia (BG), white matter perivascular spaces (WM-PVS), basal ganglia perivascular spaces (BG-PVS), and in PVS peripheries. ***p < 0.001 from paired t-tests

**Fig. 4:**
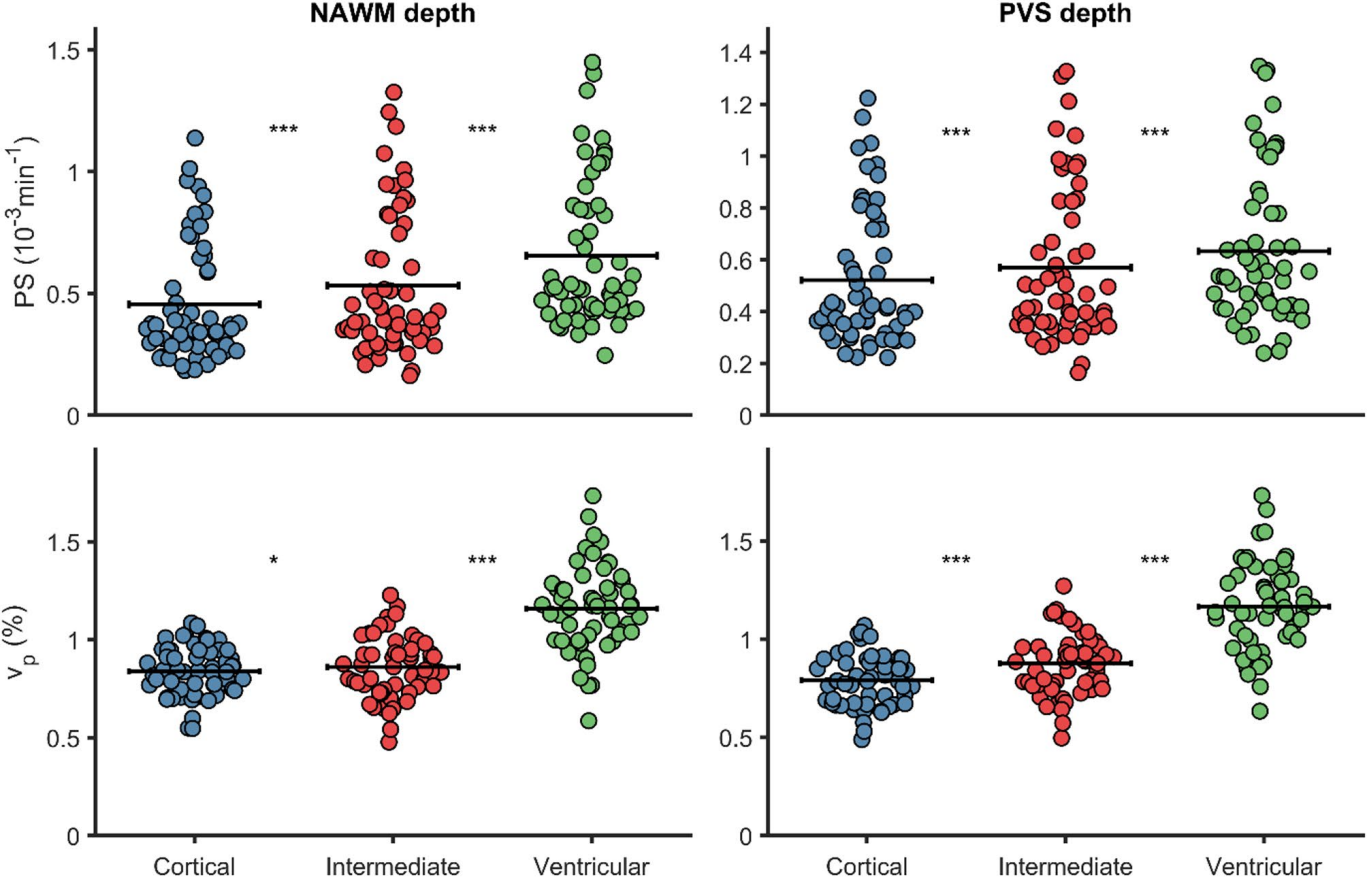
Normal appearing white matter (NAWM) and perivascular space (PVS) permeability-surface area product (PS) and fractional plasma volume ($\:{v}_{p}$) from Patlak analysis vs. white matter (WM) depth, indicating higher leakage closer to the ventricle. *p < 0.05 and ***p < 0.001 from paired t-tests (Bonferroni corrected). Statistical differences were also found using repeated measures ANOVA for NAWM depth PS (F = 163; *p* < 0.001) and $\:{v}_{p}$ (F = 306; *p* < 0.001) and for PVS depth PS (F = 66; *p* < 0.001) and $\:{v}_{p}$ (F = 293; *p* < 0.001)

Differences in PS and $\:{v}_{p}$ were compared using analysis of variance (ANOVA) (Figs. 2 and 4), paired t-tests (Figs. 2, 3 and 4), and analysis of covariance (ANCOVA) (Table 2). Pearson correlation was used to evaluate WML volume in relation to PVS volume and to evaluate WML and PVS volumes in relation to PS (Sup. Figure 4). Bonferroni correction was used to adjust for multiple comparisons where more than one paired t-test was used (Figs. 2 and 4, Sup. Figure 5, Sup. Figure 8). Bootstrapping with 1000 repetitions was used to estimate the standard deviation of the effect sizes obtained from the ANCOVA models.

## Results

### Kinetic model comparison

Before evaluating PS and $\:{v}_{p}$ in healthy tissue and vascular lesions, the Patlak model was first evaluated in relation to extended Tofts for NAWM, WML peripheries, WML, BG, WM PVS, and BG PVS. Correlations ranged between 0.38 and 0.64 for PS (all *p* < 0.01) and between 0.83 and 0.97 for $\:{v}_{p}$ for all ROIs (all *p* < 0.001), indicating that model choice has a relatively large impact on PS. Because consensus recommendations suggest using the Patlak model for subtle BBB leakage [17, 18]main analyses were first performed with the Patlak model and supplementary analyses were then performed with extended Tofts.

### Imaging markers in relation to vascular risk and global perfusion

Cohort statistics for WML volume, PVS volume, and intracranial volume (ICV) are summarized in Table 1. Further, WML and PVS volumes and PS were evaluated to vascular risk, including age, sex, hypertension and atrial fibrillation, showing higher WM PVS (*r* = 0.32; *p* = 0.02) and BG PVS (*r* = 0.31; *p* = 0.02) volumes in subject with hypertension (Sup. Table 1) but no associations to other risk factors or PS (Sup. Table 2). To test for expected associations between global perfusion and vascular density, 4D flow MRI derived total cerebral blood flow [28] was evaluated against $\:{v}_{p}$, showing significant correlations for BG (*r* = 0.30; *p* = 0.03), NAWM (*r* = 0.29; *p* = 0.03) and WM PVS (*r* = 0.28, *p* = 0.04) but not for WML (*p* = 0.96) or BG PVS (*p* = 0.32).

### Blood-brain barrier leakage within and in proximity of white matter lesions

A progressive increase in PS was observed when comparing NAWM, WML peripheries, and WML ROIs (Fig. 2), with the lowest PS in NAWM (0.49$\:\cdot\:$10^−3^$\:\cdot\:$ min^− 1^), a higher PS in WML peripheries (0.65$\:\cdot\:$10^−3^$\:\cdot\:$ min^− [1]^), and the highest PS within the WMLs (0.85$\:\cdot\:$10^−3^$\:\cdot\:$ min^− [1]^). Constraining WML ROIs to reduce CSF contamination (excluding WML voxels with CSF probability > 0.05 or T1 > 4000 ms) yielded similar results, still indicating higher PS in WMLs compared to WML peripheries (0.75$\:\cdot\:$10^−3^$\:\cdot\:$ min^− 1^ vs. 0.65$\:\cdot\:$10^−3^$\:\cdot\:$ min^− 1^; *P* < 0.001). Similar differences were also seen for $\:{v}_{p}$ among NAWM (0.88%), WML peripheries (0.99%) and WML (1.23%).

### Blood-brain barrier leakage and perivascular spaces

PS differences were also observed between healthy tissue and PVS (Fig. 3), between NAWM and WM-PVS (0.49 vs. 0.55; *p* < 0.001) and between BG and BG-PVS (0.56 vs. 0.71; *p* < 0.001). However, $\:{v}_{p}$ differences were slightly higher in NAWM compared to WM-PVS (0.88% vs. 0.86%; *p* < 0.001). In contrast, $\:{v}_{p}$ was substantially higher in BG-PVS compared to BG (1.90% vs. 1.49%; *p* < 0.001), suggesting that PS differences observed between BG-PVS and BG could be partially explained by more vasculature in the PVS.

PVS volume was also found to be highly dependent on the specific threshold, showing an exponential-like decay with increasing threshold for both WM- and BG-PVS (Sup. Figure 3). Hence, to assure that our evaluation of BBB leakage in PVS compared to NAWM and BG regions were not too threshold-sensitive, we evaluated PVS PS and $\:{v}_{p}$ as a function of PVS threshold. These results showed a weak PS-threshold dependency for WM-PVS and a slightly higher dependency for BG-PVS. Similar threshold dependencies were observed for $\:{v}_{p}$, again with a weaker dependency for WM-PVS and a more pronounced dependency for BG-PVS (Sup. Figure 3). These findings indicate that although PVS volume is highly sensitive to PVS threshold, PS and $\:{v}_{p}$ differences between tissue and PVS (Fig. 3) are not explained by PVS threshold.

### Blood-brain barrier leakage as a function of white matter depth (cortex-to-ventricle distance)

In addition to analyzing leakage within and in proximity of WML and PVS, we computed depth maps inspired by a bullseye WM parcellation [12]. Specifically, we computed cortex-to-ventricle normalized distance maps for NAWM and PVS (methods section: WM depth). Interestingly, when analyzing NAWM and PVS leakage rates, similar patterns were observed: both NAWM-PS and PVS-PS estimates were clearly higher closer to the ventricles and lower closer to the cortical boundary, with intermediate PS-values in the intermediate WM region (Fig. 4). However, similar depth-dependent patterns were also observed for v_p_(Fig. 4), suggesting that increased leakage rates as a function of WM depth are potentially explained by more vasculature.

### Separating the effects of vascular density and ROI and BBB permeability

**Fig. 5:**
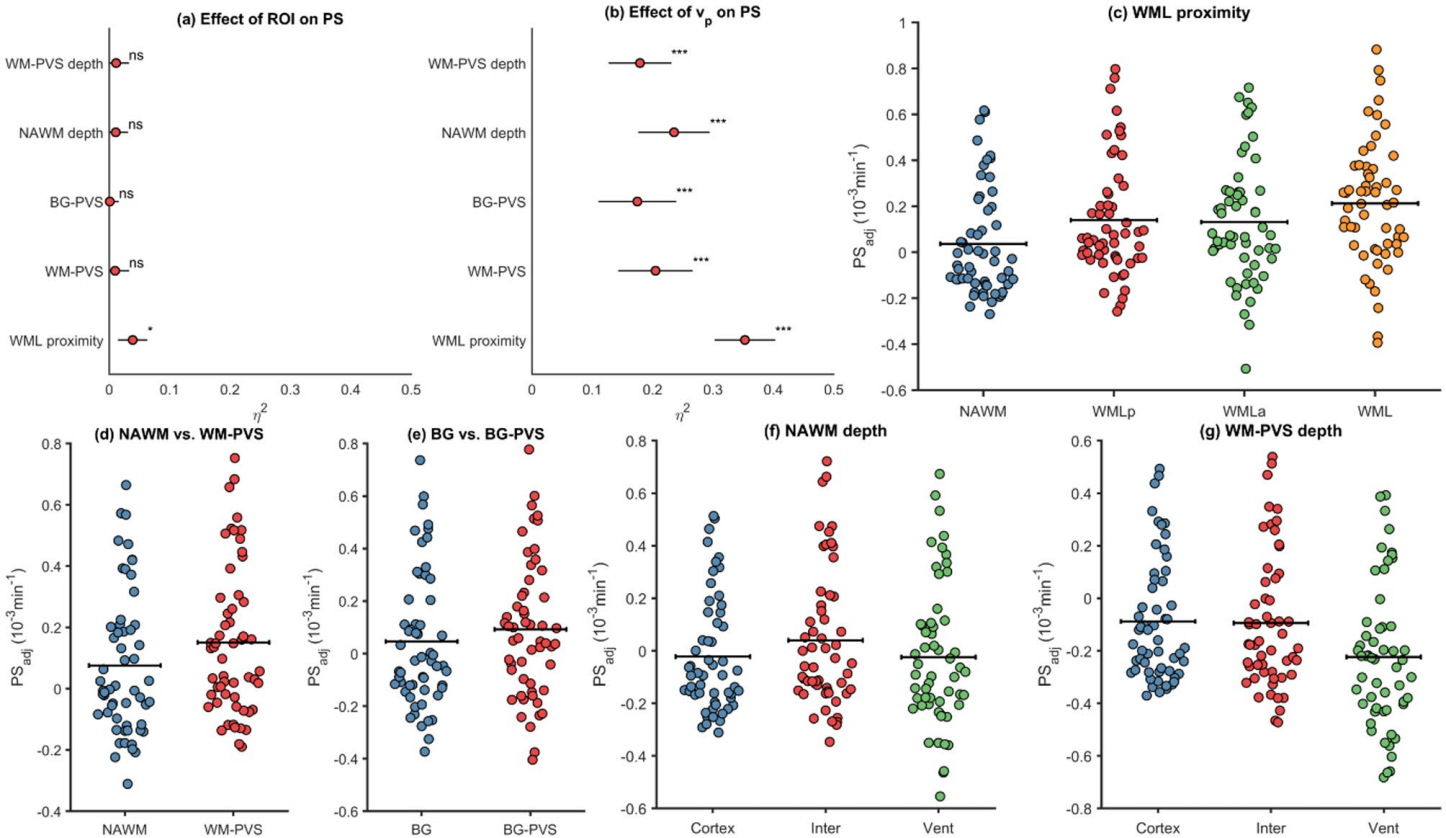
**(a-b)** Effect size ($\:\eta\:$^2^) of region-of-interest (ROI) and fractional plasma volume ($\:{v}_{p}$) on the permeability-surface area product (PS) from Patlak analysis, where the standard deviation lines were obtained by bootstrapping (*n* = 1000 samples). **(c-g)** ROI-differences in PS after removing the effect of $\:{v}_{p}$ by multiple linear regression to simultaneously fit the effects of ROI and $\:{v}_{p}$ on PS. The $\:{v}_{p}$-adjusted PS was defined by adding the mean-effect of ROI on PS to the residuals from the regression. Note that these scatter plots **(c-g)** were intended for visualization only, and statistical significance was determined entirely on the ANCOVA results in panels **(a-b)** and Table 2

Considering that stepwise increases in both PS and $\:{v}_{p}$ were observed from NAWM to WML peripheries to WML (Fig. 2), from BG to BG-PVS (Fig. 3) and from near-cortical WM and WM-PVS to periventricular WM and WM-PVS (Fig. 4), PS-rates were evaluated in ANCOVA analyses using ROI as between-factor and $\:{v}_{p}$ as covariate. Importantly, although PS-differences were still observed between NAWM, WML peripheries and WML, the effect size of ROI was weak (η^2^= 0.039; *p* = 0.03) compared to effect of v_p_ (η^2^= 0.35; *p* < 0.001). For the other analyses, no significant effect of ROI was found (> 0.29) whereas $\:{v}_{p}$ had a pronounced effect on PS (η^2^= 0.17 to η^2^= 0.35; all *p* < 0.001) in all models (Table 2; Fig. 5a-b). Further, to assess the effect of vascular risk (age, gender, hypertension, atrial fibrillation), additional ANCOVA models with both $\:{v}_{p}$ and risk factors as covariates were evaluated. Importantly, the effects of ROI and $\:{v}_{p}$ were highly similar in the adjusted models (Sup. Table 3), suggesting that our main findings (Table 2) are not confounded by vascular risk.

**Table 2:** ANCOVA to evaluate the effects of region of interest (ROI) on the permeability-surface area product (PS) in white matter (WM) and basal ganglia (BG), using the fractional plasma volume ($\:{v}_{p}$) as a covariate

	Effect of ROI	Effect of $\:{\varvec{v}}_{\varvec{p}}$
NAWM/pWML/WML*	$\:\eta\:$2 = 0.039 (*p* = 0.03)	$\:\eta\:$2 = 0.35 (*p* < 0.001)
NAWM distance**	$\:\eta\:$2 = 0.011 (*p* = 0.39)	$\:\eta\:$2 = 0.23 (*p* < 0.001)
WM-PVS distance**	$\:\eta\:$2 = 0.012 (*p* = 0.37)	$\:\eta\:$2 = 0.17 (*p* < 0.001)
NAWM/WM-PVS	$\:\eta\:$2 = 0.010 (*p* = 0.30)	$\:\eta\:$2 = 0.21 (*p* < 0.001)
BG/BG-PVS	$\:\eta\:$2 = 0.001 (*p* = 0.77)	$\:\eta\:$2 = 0.18 (*p* < 0.001)

*PS-differences between normal-appearing white matter, white matter lesion peripheries (pWML), and WML and **as a function of normalized cortex-to-ventricular distance (near-cortical WM vs. intermediate WM vs. periventricular WM)

The ANCOVA analyses were also repeated as a function of PVS threshold (Sup. Figure 4), confirming that PS differences between healthy tissue and PVS is mainly explained by $\:{v}_{p}$ independent of PVS threshold. Since most of the variance in PS was explained by $\:{v}_{p}$ rather than ROI (Table 2; Fig. 5a-b), ROI-differences in PS were visualized after adjusting for v_p_ (Fig. 5c-g). In contrast to the unadjusted plots displaying stepwise increases in PS from NAWM to WML (Fig. 2) and from near-cortical to periventricular WM (Fig. 4), only minor differences were observed from WML peripheries to WML (Fig. 5c), and near-cortical NAWM and WM-PVS showed higher $\:{v}_{p}$-adjusted PS-values than the periventricular region (Fig. 5f-g).

### Leakage rates in relation to WML and PVS volumes

Following the analyses of regional differences in PS and $\:{v}_{p}$, we evaluated relations between WML and PVS volumes, and NAWM and BG PS rates against WML and PVS volumes. While WM PVS volumes were moderately correlated with WML volume (*r* = 0.57; *p* < 0.001) and BG PVS volume (*r* = 0.54; *p* < 0.001), PS in both NAWM and BG were unrelated to WML and PVS volumes, although a weak trend (*r* = 0.13; *p* = 0.35) was found between NAWM PS and WML volume (Sup. Figure 5).

### Repeated analyses using the extended Tofts model

To assure PS and $\:{v}_{p}$ differences were not explained by model selection, all main analyses were repeated using the extended Tofts model (Sup. Figure 6–8; Sup. Table 2). Overall, the extended Tofts model led to higher PS for all ROIs compared to the Patlak model. When repeating the NAWM to WML differences, the pattern of stepwise increases in PS and $\:{v}_{p}$ remained similar for extended Tofts (Sup. Figure 6) and Patlak (Fig. 2). However, for PVS analyses, WM PVS no longer shower higher PS compared to NAWM (Sup. Figure 7). Hence, the relatively weak PS difference between NAWM and WM PVS from the Patlak model (Fig. 3) is difficult to interpret. The pattern of increased PS as a function of WM depth (Fig. 4) was relatively consistent for extended Tofts, although cortical-intermediate $\:{v}_{p}$ difference was non-significant for NAWM depth (Sup. Figure 8). Like for the Patlak model (Fig. 4), extended Tofts also showed regional differences in both PS and $\:{v}_{p}$ when comparing NAWM depth and WM PVS depth differences (Sup. Figure 8). However, the ANCOVA analyses comparing ROI differences in PS with $\:{v}_{p}$ as a covariate found no effect of ROI on PS for extended Tofts (Sup. Table 2), and the effect of $\:{v}_{p}$ on PS was also weaker compared to when using the Patlak model (Table 2), highlighting the importance of model selection for subtle BBB leakage analysis. Finally, we evaluated PS vs. $\:{v}_{p}$ and found moderate correlations for the Patlak model for all ROIs (*r* = 0.34 to *r* = 0.56; all *p* < 0.01), whereas only WML (*r* = 0.32; *p* = 0.02) showed a positive correlation for extended Tofts (Sup. Figure 9), suggesting that if PS is to be interpreted as permeability, the Patlak model might be more biased by $\:{v}_{p}$.

## Discussion

We used DCE MRI to analyze PS and $\:{v}_{p}$ in healthy tissue and within and in proximity of SVD-related lesions, to shed further light on the role of microvascular dysfunction in aging and SVD. Stepwise increases in PS and $\:{v}_{p}$ were observed from NAWM to WML peripheries to WML and from BG to BG-PVS, and PS increases were also seen from NAWM to WM-PVS. Similar increases in PS and $\:{v}_{p}$ were also found going from near-cortical to periventricular NAWM and WM-PVS. However, secondary analyses using ANCOVA to assess ROI-differences in PS with $\:{v}_{p}$ as a covariate suggested that high PS within and in proximity of SVD-features and in periventricular WM was almost entirely explained by $\:{v}_{p}$ rather than ROI, WM depth or lesion-proximity.

**Fig. 6:**
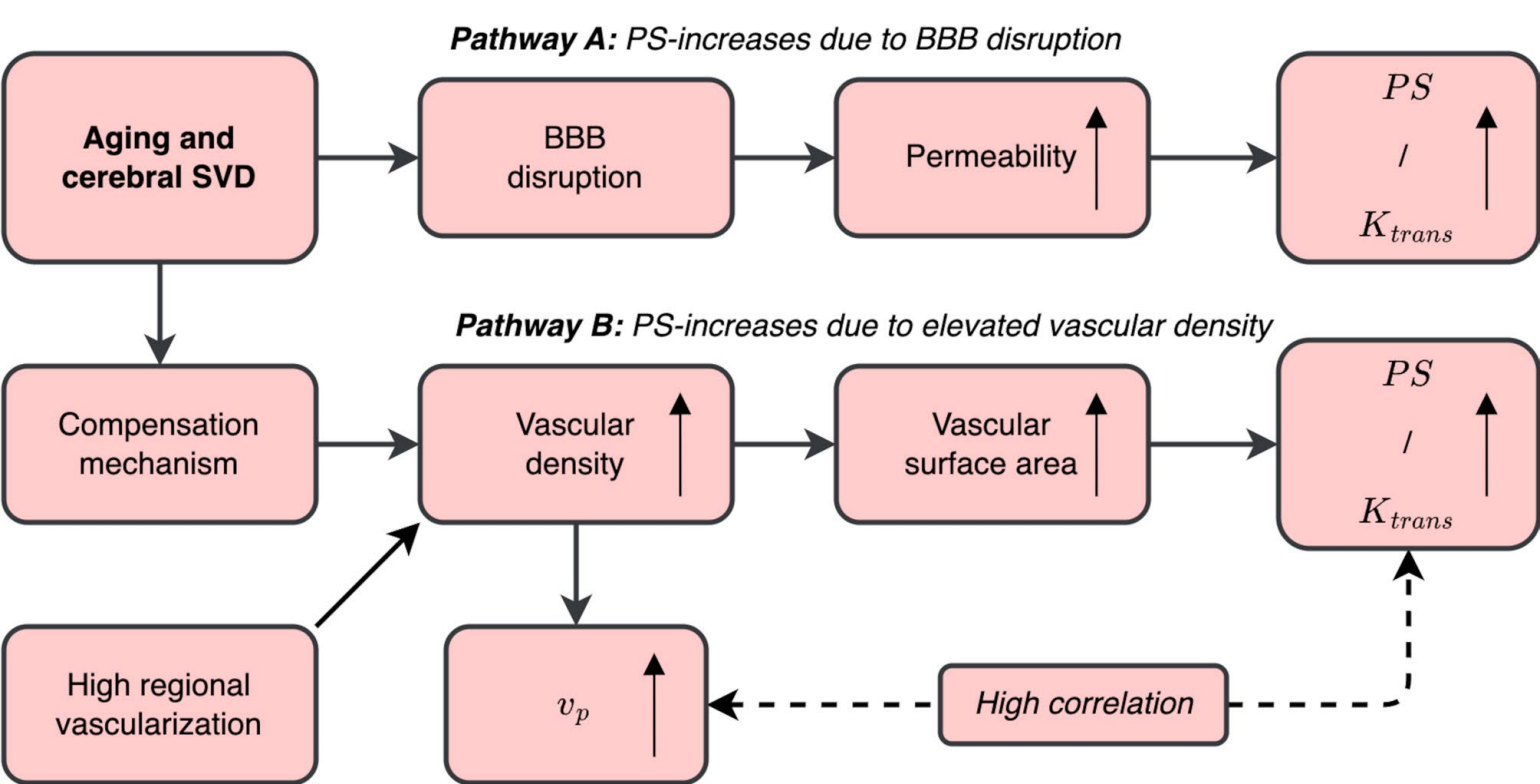
Hypothetical pathways that influence the blood-brain barrier (BBB) permeability-surface area product (PS) in aging and small vessel disease (SVD). Since PS reflects the product of BBB permeability (P) and surface area (S), high PS in a region could reflect (**A**) high permeability, indicating elevated BBB leakage, or (**B**) high microvascular density, leading to local increases in vascular surface area. If PS-increases completely reflect BBB disruption (as in pathway A), PS-differences should be independent of vascular plasma fraction ($\:{v}_{p}$), here used as a proxy for vascular surface area. On the other hand, if PS-increases are observed due to altered microvascular structure and elevated vascular surface area (as in pathway B), PS-differences should be largely explained by $\:{v}_{p}$, and a high correlation between PS and $\:{v}_{p}$ should be expected. In the latter case (**B**), high vascular density can potentially be explained by an already dense vascular network in lesion-sensitive regions, or through a compensation mechanism, where for instance hypoxia-induced vascular remodeling could lead to local increases in microvascular surface density and surface area.

Previous studies have linked BBB leakage rates to total SVD burden [15] and enlarged BG (but not WM) PVS volumes [16]and shown that NAWM PS is elevated in lacunar stroke [29, 30] and leukoaraiosis [30]. Further, a study using mixed effects models to model region-specific and patient-specific contributions to signal enhancement observed that NAWM-PS increased with reduced distance to WMLs [11] in patients with VaD. Moreover, a recent study using Patlak analysis found increased BBB leakage in WML compared to NAWM, and identified leakage hotspots in the borders of WMLs and lacunes in SVD patients [31]. Our findings using Patlak analysis to quantify the BBB leakage rate support the notion that BBB leakages are implicated in WMLs, and that the leakage rate increases with WML proximity among elderly in the population. In addition, we showed that NAWM leakage rates increase as a function of WM depth, when comparing NAWM leakage rates near the cortical boundary, in an intermediate region, and in periventricular WM. However, secondary analyses from ANCOVA to model PS as a function of ROI and $\:{v}_{p}$ do not necessarily suggest that perilesional or periventricular WM is susceptible to BBB dysfunction. Instead, the high variance explained by $\:{v}_{p}$ and the relatively small variance explained by ROI suggest that contrast accumulation in these regions could mainly reflect high vascular density rather than BBB breakdown. These findings highlight the importance of recognizing that PS (or $\:{K}_{trans}$) reflects the product of permeability (P) and surface area (S), making it impossible to distinguish between BBB permeability and leakage area. Nevertheless, NAWM to WML differences in PS were still significant after adjusting for $\:{v}_{p}$ with ANCOVA. Hence, while our findings suggest that vascular density (not ROI) explains most variance in PS across regions, they still support that BBB dysfunction is to some degree implicated in WMLs. Importantly, compartment models for IV-injections do not recognize the role of contrast clearance, further complicating the situation [32]. Hence, in future studies relying on compartment models to study subtle BBB leakages, caution is needed when interpreting high PS (or $\:{K}_{trans}$) as BBB dysfunction (Fig. 6). Here, extended Tofts was used in addition to Patlak, adding an exponential decay term that potentially could capture tracer clearance, although the intention is to capture backflow to the plasma compartment [17]. In our case, most results remained similar with extended Tofts, although the effects of both ROI and $\:{v}_{p}$ on PS were weaker compared to the Patlak model. Importantly, while our results show that PS from extended Tofts might also be less biased by confounding vascular density compared to Patlak, the effect of ROI on PS was non-significant in all ANCOVA models when adjusting for $\:{v}_{p}$ in the extended Tofts results.

Importantly, we do not have the ability to assess vascular remodeling with the cross-sectional design of the current study, making our proposed hypothesis (Fig. 6) highly speculative at this point. However, hypoxia induces angiogenic factors in microvascular endothelium [33] and elevates cerebral microvascular density in rats [34–36] which is more pronounced in deep WM [37]. In humans, angiogenic factors are linked to SVD burden [38, 39] and PET imaging of angiogenesis shows elevated levels in the WML penumbra [40]. While a compensation mechanism (an angiogenic response) to insufficient hemodynamics may increase perfusion, it could also elevate BBB leakage and inflammation [41, 42]. However, the WML core is characterized by hypoperfusion [43, 44]suggesting that the angiogenic response could partially be maladaptive. Nevertheless, our proposed hypothesis needs validation in future work. Potentially, high $\:{v}_{p}$ and PS could also be explained by the spatial distribution of vasculature in the brain. For instance, the venular vasculature could intrinsically be more dense in periventricular WM where WMLs typically develop. Interestingly, a recent MRI approach combining susceptibility weighted imaging (SWI) and fluid attenuated inversion recovery (FLAIR) scans (SWI-FLAIR fusion), enhanced by Ferumoxytol contrast to achieve vessel imaging at extremely high in-plane resolution (0.22 × 0.22mm [2]), described vascular remodeling and excessively high venous density within and in proximity of vascular lesions [45]. While the acquired DCE resolution of the current study (1.5 mm isotropic) limits our ability to investigate venous vascularization patterns in similar detail, vascular remodeling on the venous side could potentially explain the patterns of high $\:{v}_{p}$ and PS observed in our study within WML, PVS and in periventricular NAWM. Recently, Voorter et al. [44] evaluated pathophysiological heterogeneity of deep and periventricular WML by combination of FLAIR, DCE MRI and diffusion-weighted MRI [44]. While the authors found differences in diffusivity and water content between the WML types, no differences were found in perfusion and BBB leakage [44]. Further, while their spatial analysis showed significant gradients in WM microstructure, water content, perfusion and $\:{v}_{p}$ within and in WML peripheries, such differences were not observed for PS [44]. Importantly, longitudinal population-based studies are needed to investigate whether NAWM leakage rates at baseline may predict WML and PVS progression over time. Such studies could provide important insights into the temporal dynamics among BBB dysfunction and the progression of more pronounced SVD-related lesions, e.g., WML and dilated PVS, which is critical to better understand the pathophysiology of SVD. Furthermore, increasing evidence highlight the role of neuroinflammation in WML progression [46–48]highlighting the need for joint investigation of microvascular structure, BBB dysfunction and inflammatory markers.

SVD has high clinical signficiance [1] and the etiology is not entirely understood [2]. WMLs are linked to increased risk for stroke, hemorrhage, dementia and death [1]. Radiological hallmarks of SVD are common in the population [49]and both WML [6, 50] and PVS [6, 51, 52] increases longitudinally with age and vascular risk [50, 52]. In the current study, total WML and total PVS volumes were correlated, aligning with previous findings [6]. However, the BBB leakage rate in NAWM was unrelated to WML volume and WM-PVS volume, and the BG leakage rate was unrelated to BG-PVS volume. In contrast, others have found that NAWM-PS correlates with total WML volume [15, 53]total PVS volume and total SVD score [15]and with BG (but not WM) PVS volume [16]. Importantly, while our findings that $\:{v}_{p}$ rather than ROI (WM depth or lesion-proximity) explains most variance in BBB-PS, the fact that high NAWM-PS is correlated with WML burden in previous studies [15, 16, 53] still supports a role of BBB dysfunction in SVD.

Contrast accumulation in PVS could have at least three explanations: (1) leakage through the choroid plexus, propagation through the subarachnoid space (SAS) CSF and propagation along PVS, (2) leakage across arteries residing in the SAS CSF and propagation along PVS, or (3) directly across the BBB, or a mix of all three explanations. Considering the time scale of the DCE imaging experiment (17.5 min), most of the PVS accumulation is likely explained by leakage directly across the BBB. Hence, our findings suggest that BBB leakages in dilated PVS and their surroundings are the cause of the observed Gadolinium accumulation in PVS, rather than transport from other CSF regions. Naganawa et al. [54] observed intravenous leakage to large PVS four hours after IV-injection. However, the lack of dynamic imaging in that study makes it difficult to compare the potential routes of the contrast. Regarding the PVS segmentation, previous studies using automatic methods have shown widely different results, ranging from total PVS volumes of ~ 0.3 mL [55] to WM-PVS volumes of ~ 5 mL [56] or relative WM-PVS volumes of ~ 4.9% [57] (~ 20 mL assuming 400 mL WM volume). Hence, we evaluated PVS volumes, PS, and $\:{v}_{p}$ as a function of PVS threshold. We demonstrated that total PVS volume and PVS volume fraction both decayed exponentially as the PVS threshold was increased, and that the estimation of WM-PVS volume decreases dramatically from a 5% (11.6 mL) to 25% threshold (0.11 mL). Like many other PVS studies [58] we applied a hessian-based filter [21] to boost PVS visualization before thresholding. This approach was evaluated previously, demonstrating good agreement between cerebral WM-PVS volumes and manual PVS counts in the semioval center [6].

Regarding limitations, our MRI protocol did not allow separation of arterial/venous PVS. Other studies using 7T systems have identified arteriolar PVS using a time-of-flight scan sensitive to the inflow effect [59] or venular PVS using susceptibility weighted imaging sensitive to deoxygenated blood [60]. Based on such studies and on other observations, MRI-detectable PVS are considered to be almost exclusively arteriolar [5, 59, 60]. Considering that Buch et al. [45] observed high venous density in proximity of vascular lesions, it is plausible that differences between tissue and PVS would have been more pronounced around venous PVS. Further, the PVS voxels likely reflect a combination of vessel, CSF (i.e., the true “PVS”) and tissue, which is a limitation of the spatial resolution. This may explain why PS and $\:{v}_{p}$ did not differ dramatically between WM and WM PVS, whereas pronounced differences were observed between BG and BG PVS for both PS and $\:{v}_{p}$, since the segmented BG-visible PVS ROIs likely correspond to larger vessels and CSF spaces and less tissue. Moreover, if contrast accumulation in PVS is mainly due to leakage across the BBB, arteriolar and venular PVS would show similar concentration curves when using a frame rate of 17.5s (as the current study), assuming a cerebral circulation time of 5–6 seconds [61, 62] and an arterial transit time of 600–1500 ms [63, 64]. Furthermore, WMLs were defined as T1-weighted hypointensities obtained from the FreeSurfer segmentations. Hence, no FLAIR scan was used to distinguish WMLs from CSF. However, T1-hypointensities have shown good agreement with Fazekas score [65]and our finding of higher BBB leakage rates in WML compared to NAWM and WML peripheries was still pronounced when including T1 time and CSF probability constraints to the WML segmentations, ensuring that the WML concentration curves are not biased by CSF inclusion. Finally, spatiotemporal artifacts and subject motion could give rise to false PVS [66] and BBB leakages [67]. However, our T1-weighted scans appeared relatively free from Gibbs ringing, and a post-processing filter [23] was applied to the DCE data prior to co-registration to minimize the impact of Gibbs artifacts on BBB leakage estimations.

In conclusion, BBB leakage rates increased from NAWM to WMLs, from NAWM and BG to PVS, and from near-cortical to periventricular WM, findings that if considered in isolation, would align with BBB dysfunction in SVD. However, high PS was mainly explained by high $\:{v}_{p}$ rather than lesion-proximity, suggesting high vascular density and not necessarily BBB dysfunction in perilesional and periventricular tissue may have contributed to previous observations. Collectively, our findings highlight the importance of recognizing PS (or $\:{K}_{trans}$) as the product of permeability and vascular surface area, and that new compartment models that better incorporate differences in both vascular density and potential clearance of contrast over time are needed to understand the role of BBB dysfunction in aging and SVD.

## Electronic supplementary material

Below is the link to the electronic supplementary material.

Supplementary Material 1

## Data Availability

The datasets used and/or analyzed during the current study are available from the corresponding author on reasonable request.

## Abbreviations

ANOVA/ANCOVA: Analysis of variance/analysis of covariance
BBB: Blood-brain barrier
BG: Basal ganglia
CSF: Cerebrospinal fluid
DCE MRI: Dynamic contrast enhanced magnetic resonance imaging
GM: Gray matter
MoCA: Montreal cognitive assessment score
MRI: Magnetic resonance imaging
NAWM: Normal-appearing white matter (white matter free from any MRI-visible lesions)
PS: Permeability-surface area product (blood-brain barrier leakage rate from the Patlak model)
PVS: Perivascular spaces
ROI: Region of interest
SVD: Cerebral small vessel disease
VFA: Variable flip angle
VIF: Vascular input function
υ_p_: Fractional plasma volume (estimated fraction of a voxel containing blood plasma from the Patlak model)
WM: White matter
WML: White matter lesions
