# Supplementary material for: MRI contrast accumulation in features of cerebral small vessel disease: blood-brain barrier dysfunction or elevated vascular density?
Source: Fluids Barriers CNS. 2025 Jul 16;22:74. doi: 10.1186/s12987-025-00675-4 (PMC12265124; doi:10.1186/s12987-025-00675-4)
Supplement: Supplementary file 1 — Supplementary Material 1 [file 12987_2025_675_MOESM1_ESM.pdf]

Supplementary information for:

MRI contrast accumulation in features of cerebral small vessel disease: blood-brain barrier dysfunction or elevated vascular density?

Authors: Tomas Vikner[1,2,3], Anders Garpebring[1], Cecilia Björnfot[1,4], Jan Malm[5], Anders Eklund[1,2], and Anders Wåhlin[1,2,4]

1. Department of Diagnostics and Intervention, Umeå University, S-90187 Umeå, Sweden

2. Department of Applied Physics and Electronics, Umeå University, S-90187 Umeå, Sweden

3. Department of Medical Physics, School of Medicine and Public Health, University of Wisconsin-Madison, Madison, WI, 53792, USA

4. Umeå Center for Functional Brain Imaging (UFBI), Umeå University, S-90187 Umeå, Sweden

5. Department of Clinical Science, Neurosciences, Umeå University, S-90187 Umeå, Sweden

Corresponding authors:

Tomas Vikner (tomas.vikner@umu.se) +1608 514 5170

Department of Diagnostics and Intervention, Umeå University, SE 901 87 Umeå, Sweden

Anders Wåhlin (anders.wahlin@umu.se) +4670 253 7426

Department of Applied Physics and Electronics, Umeå University, SE 901 87 Umeå, Sweden

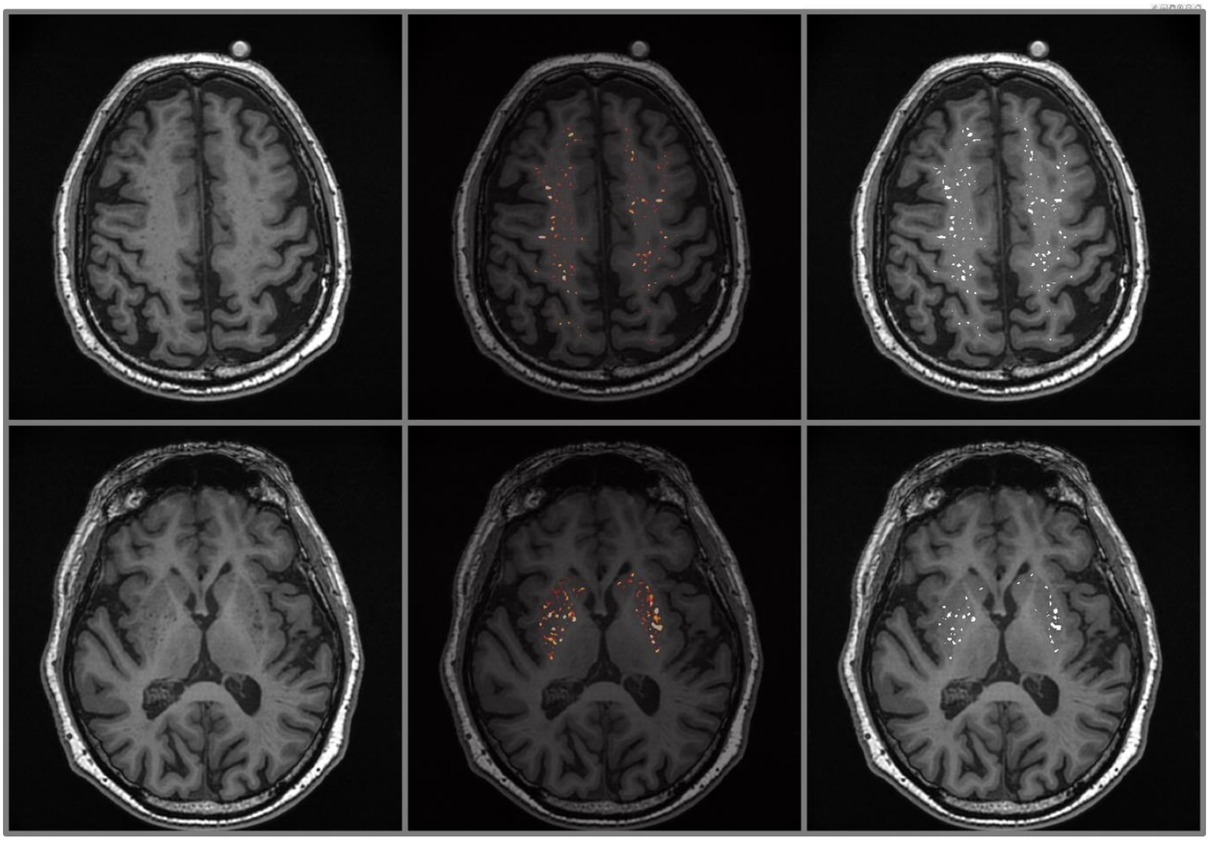

Sup. Fig. 1. Perivascular spaces in white matter (WM; top row) and basal ganglia (BG; lower row). The middle column corresponds to PVS enhancement maps obtained by applying hessian-based filter (Jerman et al.) to the T1-weighted volume (left). Binary PVS volumes (right) were obtained using 5% WM PVS threshold and a 15% BG PVS threshold to the filtered T1-weighted scans (middle).

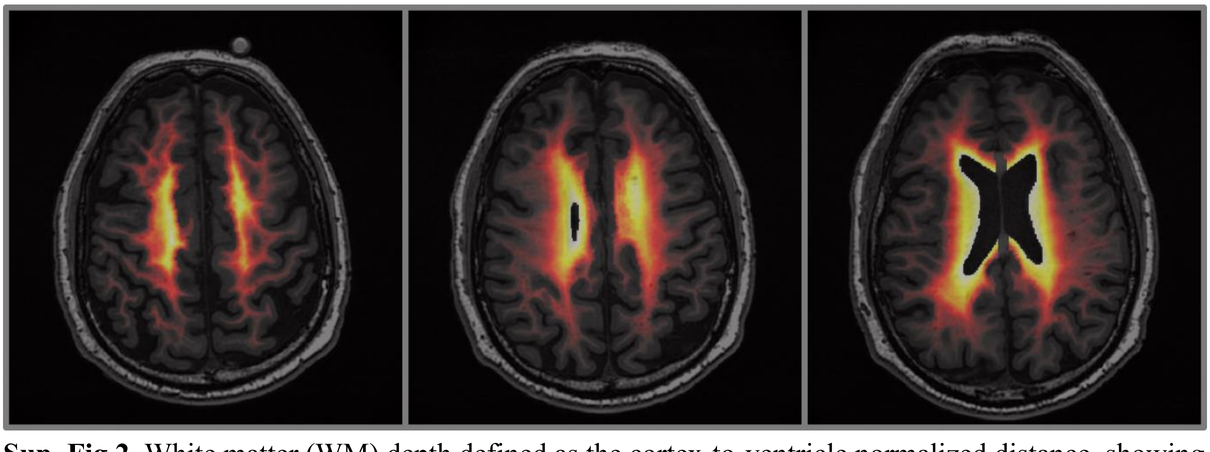

Sup. Fig 2. White matter (WM) depth defined as the cortex-to-ventricle normalized distance, showing low values (approach 0) near the cortical boundary and high values (approaching 1) near the ventricular boundary.

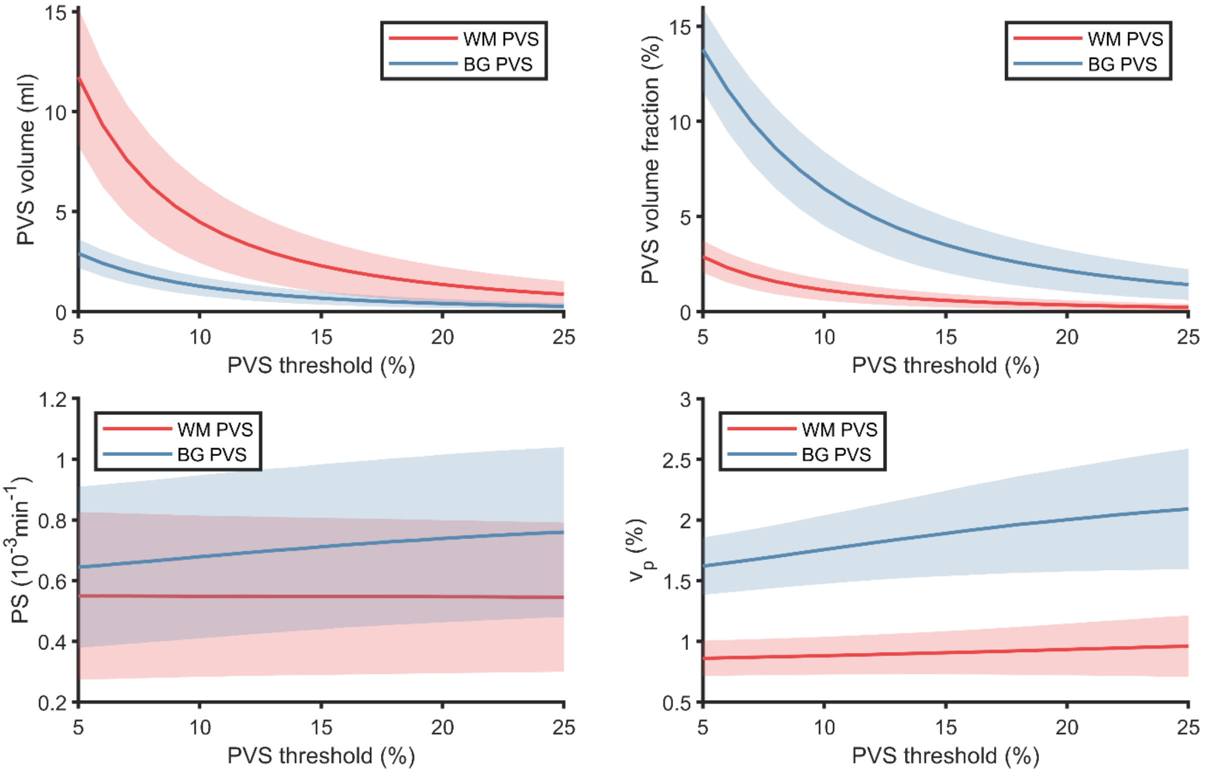

Sup. Fig. 3. Cerebral white matter (WM) and basal ganglia (BG) perivascular space (PVS) volume, volume fraction (%), permeability-surface area product (PS), and fractional plasma volume (v_p) as a function of PVS threshold, indicating slight PS increases with higher PVS thresholds. Shaded regions correspond to ±1 standard deviation.

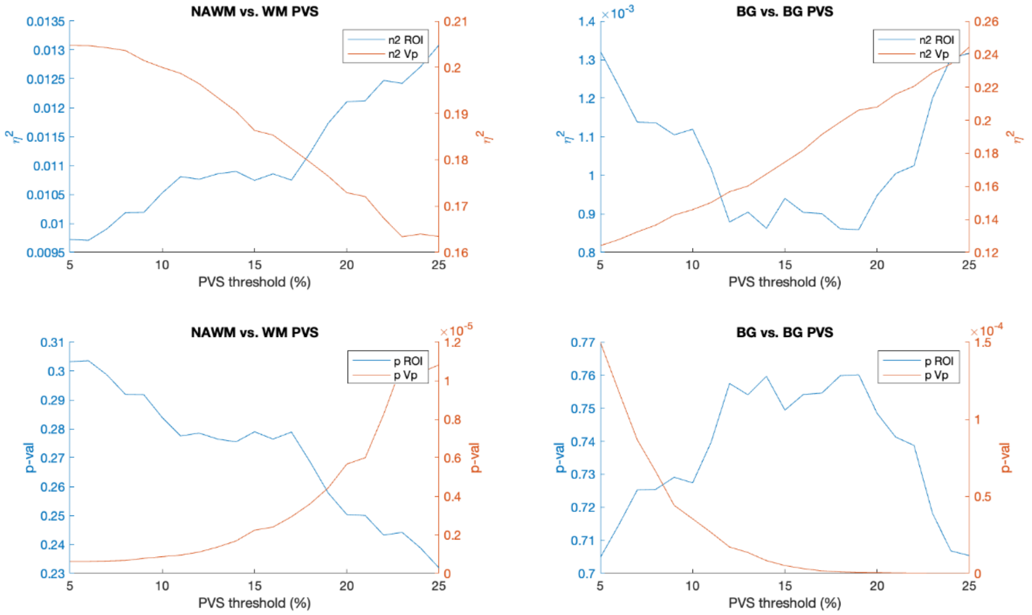

Sup. Fig. 4. ANCOVA to test the effects of ROI and fractional plasma volume (v_p) on the permeability-surface area product (PS) for a range of PVS thresholds. Effect sizes (η^2) and p-values from ANCOVA models show a significant contribution of v_p to variability in PS, whereas the effect of ROI (tissue vs. PVS) appears minimal when controlling for v_p, for all PVS thresholds.

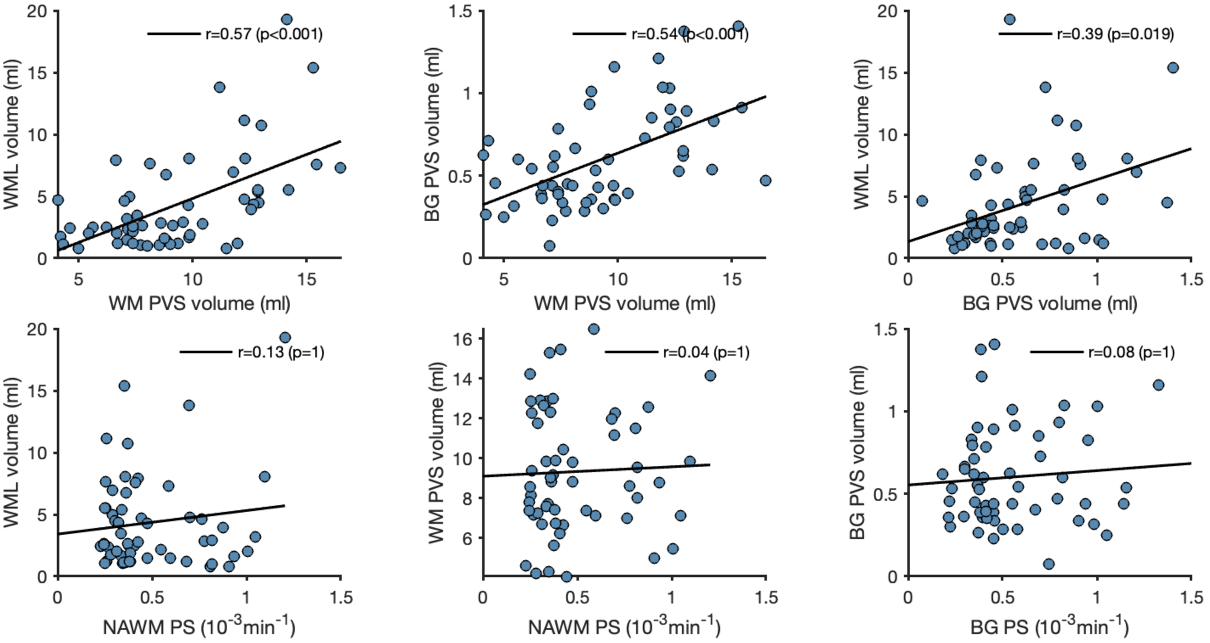

Sup. Fig. 5. White matter lesion (WML) volume in relation to white matter (WM) and basal ganglia (BG) perivascular space (PVS) volume, and WML and PVS volume in relation to permeability-surface area product (PS) in normal-appearing WM and BG. The WML volumes were transformed using the natural logarithm (ln) to achieve normality. The correlation coefficients (r) and p-values (Bonferroni corrected) were obtained from Pearson correlation.

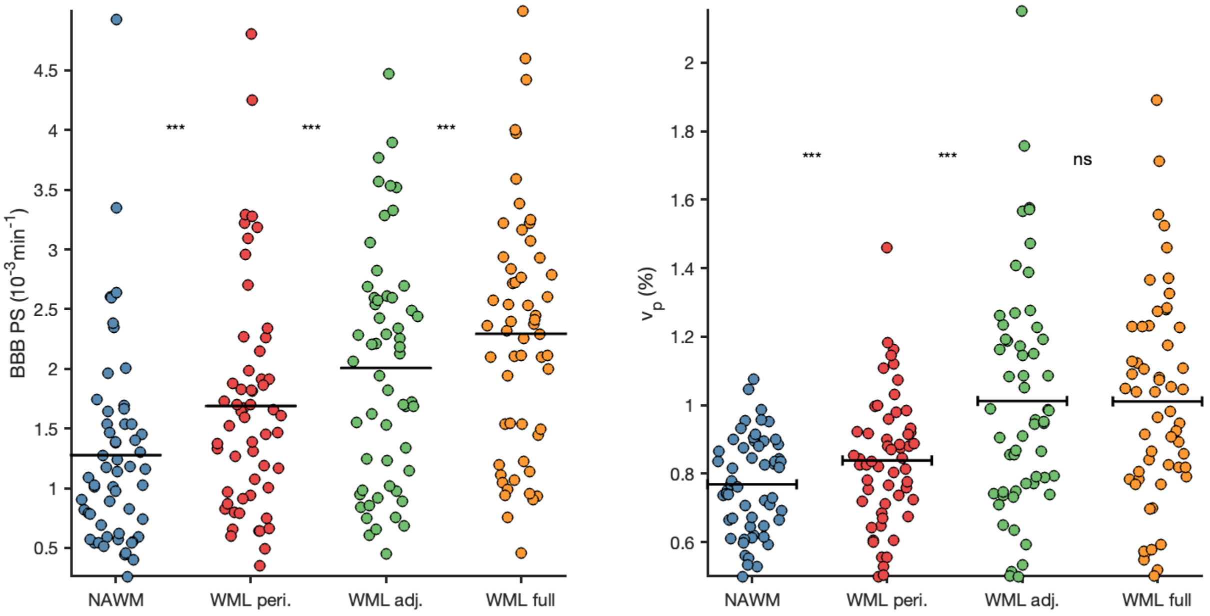

Sup. Fig. 6. Regional differences in permeability-surface area product (PS) and plasma fraction (v_p) when using extended Tofts to replicate the main results obtained from the Patlak model (Figure 2). ***p<0.001 from paired t-test (multiple comparison adj.) Statistical differences were also obtained using repeated measures ANOVA for PS (F=62; p<0.001) and v_p (F=39; p<0.001).

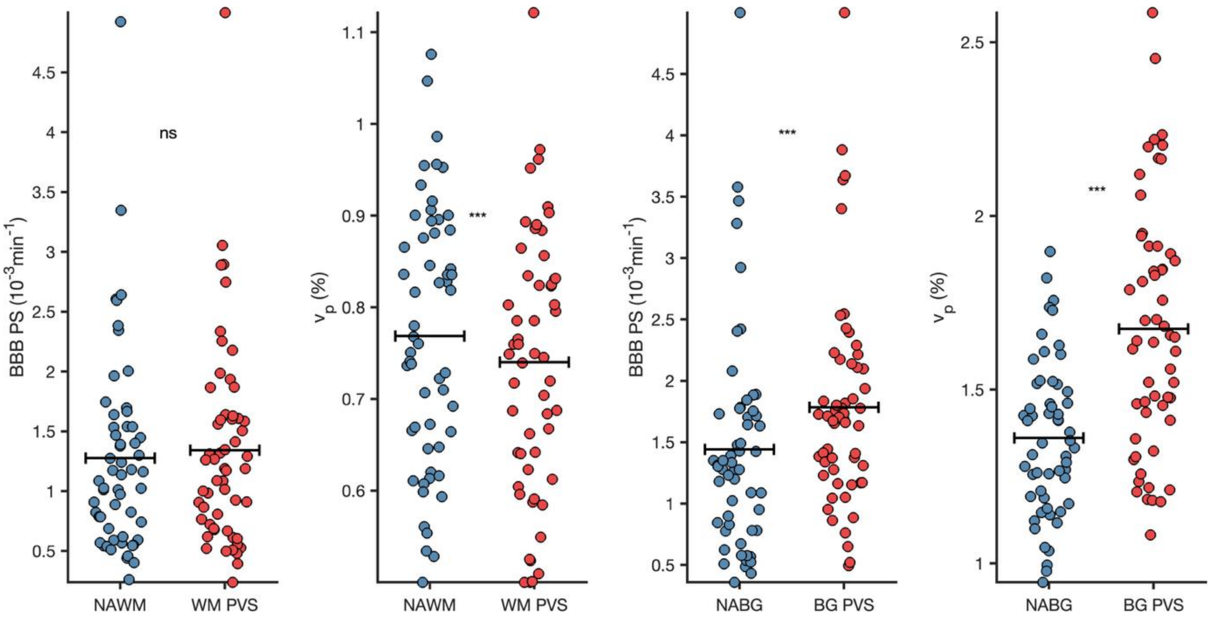

Sup. Fig. 7. Regional differences in permeability-surface area product (PS) and plasma fraction (v_p) when using extended Tofts to replicate the main results obtained from the Patlak model (Figure 3). ***p<0.001 from paired t-test.

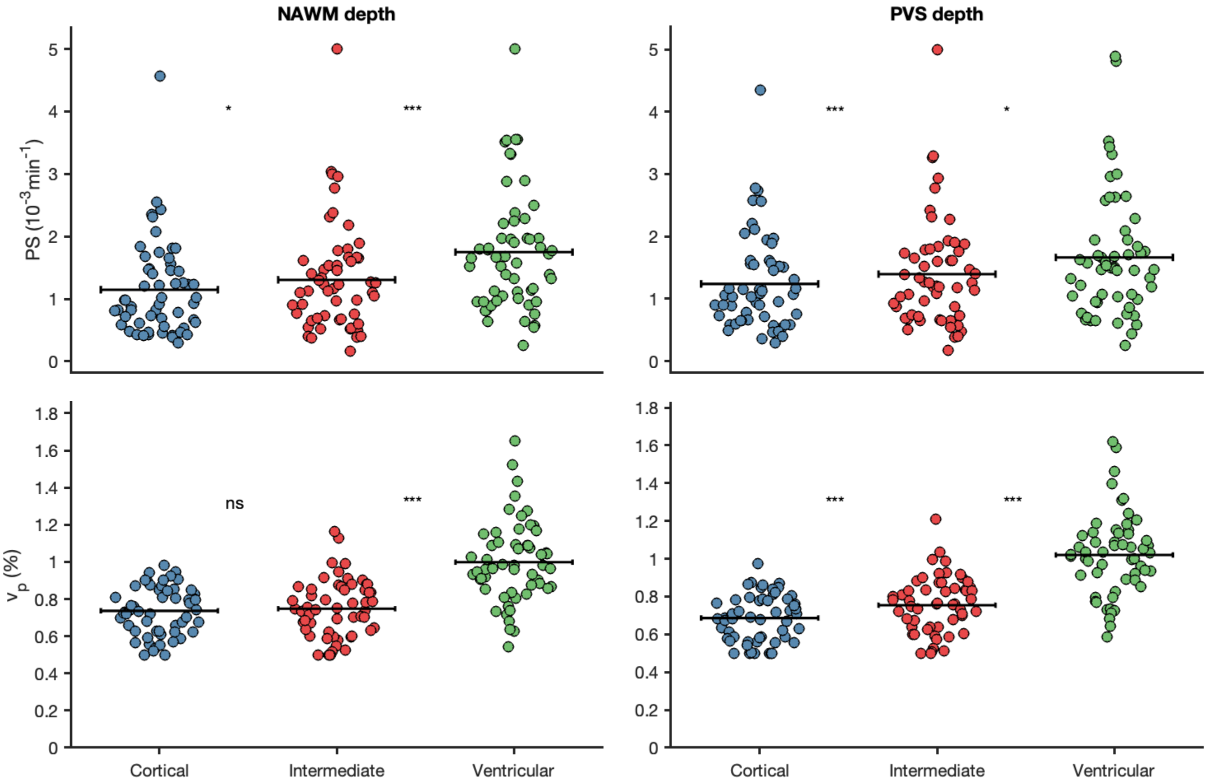

Sup. Fig. 8. Regional differences in permeability-surface area product (PS) and plasma fraction (v_p) when using extended Tofts to replicate the main results obtained from the Patlak model (Figure 4). *p<0.005 and ***p<0.001 from paired t-test (multiple comparison adj.). Statistical differences were also found using repeated measures ANOVA for NAWM depth PS (F=37; p<0.001) and v_p (F=231; p<0.001) and for PVS depth PS (F=18; p<0.001) and v_p (F=282; p<0.001).

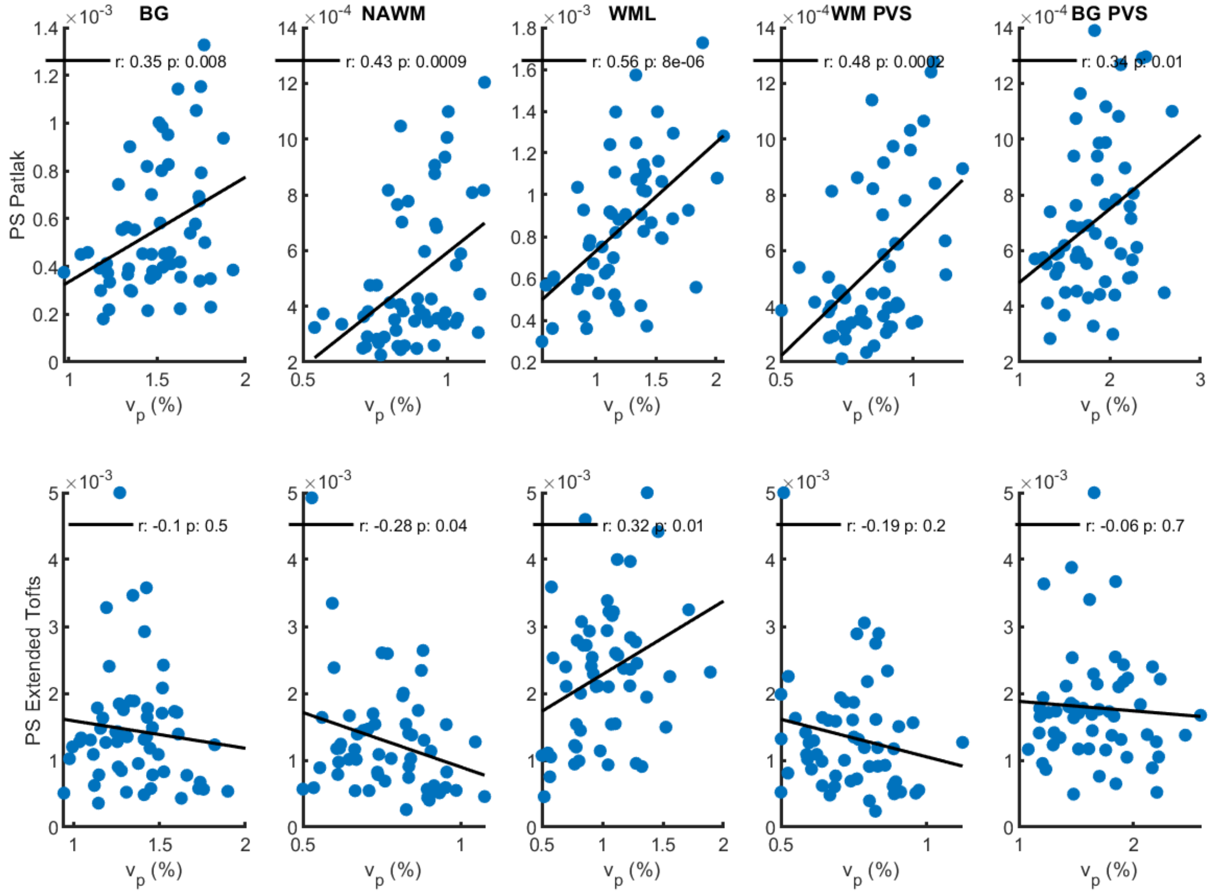

Sup. Fig. 9. Permeability-surface area product (PS) in relation plasma fraction (v_p) for basal ganglia (BG), normal-appearing white matter (NAWM), white matter lesions (WML), WM perivascular spaces (WM PVS) and BG PVS, showing correlations between PS and v_p for all ROIs when using the Patlak model, whereas only WML shows a positive association for extended Tofts.

Sup. Tab. 1.

**Imaging Marker**	**Age**	**Gender**	**Hypertension**	**Atr. Fibrillation**
WML Vol.	-0.17	0.10	0.22	0.14
WM PVS Vol.	0.03	0.10	0.32*	0.22
BG PVS Vol.	-0.19	-0.03	0.30*	0.22
BG PS	-0.24	-0.09	0.01	-0.01
NAWM PS	-0.22	0.00	-0.02	0.01
WML PS	-0.14	-0.07	-0.07	-0.05
WM PVS PS	-0.26	-0.03	0.02	0.03
BG PVS PS	-0.19	-0.10	-0.03	-0.04

Gender corresponds to male (1) and female (2). WML, white matter lesions; WM, white matter; BG, basal ganglia; PVS, perivascular space. Correlation coefficients (r) from Pearson correlation. *p<0.05. Since this evaluation was not part of our hypothesis test, no multiple comparison correction was used.

Sup. Tab. 2. ANCOVA to evaluate the effect of ROI on the permeability-surface area product (PS) in white matter (WM) and basal ganglia (BG) with plasma fraction (v_p) as covariate, for extended Tofts.

	Effect of ROI on PS	Effect of v_p on PS
NAWM/WMLP/WML*	η2=0.014 (p=0.37)	η2=0.07 (p<0.001)
NAWM distance**	η2=0.026 (p=0.11)	η2=0.001 (p=0.76)
WM-PVS distance**	η2=0.029 (p=0.09)	η2=0.001 (p=0.70)
NAWM/WM-PVS	η2=0.003 (p=0.52)	η2=0.058 (p=0.01)
BG/BG-PVS	η2=0.004 (p=0.53)	η2=0.001 (p=0.76)

Note: *PS-differences between normal-appearing white matter, white matter lesion (WML) peripheries, and WML and **as a function of normalized cortex-to-ventricular distance (near-cortical WM vs. intermediate WM vs. periventricular WM).

Sup. Tab. 3. ANCOVA to evaluate the effects of ROI on the permeability-surface area product (PS) in white matter (WM) and basal ganglia (BG) plasma fraction (v_p) with Patlak and vascular risk factors as a covariates.

	Effect of ROI on PS	Effect of v_p on PS
NAWM/WMLP/WML*	η2=0.040 (p=0.032)	η2=0.349 (p<0.001)
NAWM distance**	η2=0.011 (p=0.40)	η2=0.245 (p<0.001)
WM-PVS distance**	η2=0.012 (p=0.39)	η2=0.181 (p<0.001)
NAWM/WM-PVS	η2=0.010 (p=0.31)	η2=0.196 (p<0.001)
BG/BG-PVS	η2=0.001 (p=0.74)	η2=0.172 (p<0.001)

Note: *PS-differences between normal-appearing white matter, white matter lesion (WML) peripheries, and WML and **as a function of normalized cortex-to-ventricular distance.
